# EMMPRIN/CD147 up-regulates urokinase-type plasminogen activator: implications in oral tumor progression

**DOI:** 10.1186/1471-2407-12-115

**Published:** 2012-03-23

**Authors:** Géraldine Lescaille, Suzanne Menashi, Bénédicte Cavelier-Balloy, Farah Khayati, Cathy Quemener, Marie Pierre Podgorniak, Benyoussef Naïmi, Fabien Calvo, Céleste Lebbe, Samia Mourah

**Affiliations:** 1 Inserm, UMR-S 940, Paris F-75010, France; 2 CNRS, EAC 7149, Laboratoire CRRET, Université 12, Créteil F-94000, France; 3 Cabinet de Dermatopathologie 35, avenue Mathurin Moreau, 75019 Paris, France; 4 AP-HP, Hôpital Saint-Louis, Laboratory of Pharmacology, Paris F-75010, France; 5 Université Paris 12, Créteil, Paris, France; 6 Université Paris 7- IUH, Paris F-75010, France; 7 Département de Dermatologie, hôpital Saint Louis, Paris F-75010, France; 8 Laboratoire de Pharmacologie and INSERM U940, Hôpital Saint-Louis, 27, rue Juliette Dodu, 75010 Paris, France

**Keywords:** EMMPRIN/CD147, uPA, Oral squamous cell carcinoma, Invasion, Progression

## Abstract

**Backgrounds:**

An elevated level of EMMPRIN in cancer tissues have been correlated with tumor invasion in numerous cancers including oral cavity and larynx. Although EMMPRIN's effect has been generally attributed to its MMP inducing activity, we have previously demonstrated in breast cancer model that EMMPRIN can also enhance invasion by upregulating uPA. In this study, the role of EMMPRIN in regulating uPA and invasion was investigated in oral squamous cell carcinoma (OSCC) progression.

**Methods:**

Precancerous and invasive oral tumoral tissues were used as well as the corresponding cell lines, DOK and SCC-9 respectively. The paracrine regulation of uPA by EMMPRIN was investigated by treating culture cells with EMMPRIN-enriched membrane vesicles. UPA expression was analyzed by qPCR and immunostaining and the consequence on the invasion capacity was studied using modified Boyden chamber assay, in the presence or absence of EMMPRIN blocking antibody, the uPA inhibitor amiloride or the MMP inhibitor marimastat.

**Results:**

OSCC tumors were shown to express more EMMPRIN and uPA compared to dysplastic lesions. The corresponding cell models, SCC-9 and DOK cells, displayed similar expression pattern. In both cell types EMMPRIN upregulated the expression of uPA as well as that of MMP-2 and MMP-9. EMMPRIN treatment led to a significant increase in cell invasion both in the invasive SCC-9 and in the less invasive dysplastic DOK cells, in an MMP and uPA dependent manner.

**Conclusions:**

Our results suggest that the upregulation of uPA contributes to EMMPRIN's effect in promoting oral tumor invasion.

## Background

Oral squamous cancer cell carcinoma (OSCC) ranks among the top ten most frequently cancers, and 500 000 people per year are world widely diagnosed [1]. OSCC is highly invasive with bad prognosis; despite the recent advances in cancer therapy, the 5-year survival rate of patients has remained at < 50% [2]. Little is known about of the molecular events that govern OSCC initiation, progression and metastasis. Development of OSCC is a complex and multistep process, with transformation from oral premalignant dysplastic lesion to OSCC. Progression is generally known to involve the intervention of proteinases [3-5]. Extracellular matrix metalloproteinase inducer (EMMPRIN/CD147), a membrane glycoprotein greatly enriched on the surface of tumor cells, is mainly known for its ability to increase the synthesis of MMPs in tumor cells and in the neighbouring stromal cells, such as fibroblasts and endothelial cells [6-10]. EMMPRIN has been implicated in tumor invasion and its elevated levels in cancer tissues have been correlated with tumor progression in numerous malignant tumor models including tumors of the oral cavity and larynx [11,12].

In addition to increasing invasion through proteinase induction, EMMPRIN induces several other malignant properties associated with cancer. These include, amongst others, the stimulation of cell survival signaling, including Akt, Erk and FAK, through the increased production of the pericellular polysaccharide hyaluronan [13]. Also, EMMPRIN can promote angiogenesis by the upregulation of VEGF expression as well as its main receptor VEGFR-2 in both tumor cells and endothelial cells [14-16]. This effect on VEGF and VEGFR-2 was shown to be mediated by HIF-2α [17].

The role of EMMPRIN in tumor growth and invasion was illustrated by the accelerated growth and increased invasiveness of EMMPRIN-overexpressing human breast cancer cells [18,19]. The increased tumor size in the EMMPRIN overexpressing cells was associated with an increase, in the tumors, of not only MMP-2 and MMP-9 [18,19], but also of urokinase type plasminogen activator (uPA) levels [18]. Indeed, we have previously reported that EMMPRIN is able to upregulate the expression of the plasminogen activation system, including uPA, in mammary tumor cells, further increasing its proteolytic and invasion potential [18].

Microarray analyses of primary oral tumors have identified uPA and its receptor (uPAR) as key genes associated with human OSCC progression [18,20,21]. Human OSCC tumors with high levels of uPA and uPAR are more invasive, exhibit enhanced lymph node metastasis and more frequent tumor relapse [22].

Increased expression of EMMPRIN in oral squamous cell carcinoma has been shown to correlate with lymphatic metastasis and tumor progression [23]. EMMPRIN overexpression has been previously reported to occur at a very early stage of oral carcinogenesis and to play a contributing role in OSCC tumorogenesis [24]. Its role in facilitating tumor cell motility was attributed to its ability to increase MMP production and tenascin-C matrix deposition [25,26]. In this study using both invasive and precancerous oral cancer cell models we present evidence suggesting that EMMPRIN promotes oral tumor invasion by inducing uPA expression.

## Methods

### Cell culture

Two cell lines representing two stages of oral tumour progression were used: DOK, a precancerous dysplastic cell line [27] and SCC-9, an oral squamous carcinoma cell line (Rheinwald laboratory). The cells were cultured in DMEM with 10% **fetal bovine serum (FBS) **and 2mML-glutamine. Chinese Hamster Ovary (CHO) cells (ATCC, Rockville, MB) were cultured in DMEM/F12 (Invitrogen) supplemented with 10% FBS and 2mML-glutamine.

### Membrane preparation

CHO cells were stably transfected with a plasmid containing full-lengh EMMPRIN cDNA (CHO-Emp cells) or empty vector (CHO-Mock cells) [18]. CHO-Emp and CHO-Mock membranes were isolated by differential centrifugation as previously described [18]. The bioactivity of EMMPRIN-containing membranes was verified by its ability to stimulate uPA expression in melanoma cells [18]. The membrane vesicles obtained from the CHO-Emp or CHO-Mock cells are referred to as CHO-Emp mb or CHO mb respectively throughout.

### Small interfering RNA transfection

EMMPRIN small interfering RNA(siRNA) oligos (Ambion/Applied Biosystems) or scrambled siRNA oligos (25 nmol/L) were transfected into DOK and SCC-9 cells using the BLOCK-iT transfection kit and Lipofectamine-2000 (Invitrogen), according to the manufacturer's protocol. Cells were then incubated for 24 hours prior to the measurements of uPA mRNA by qRT-PCR and invasion assays.

### Western blotting analysis

Western blot was preformed as previously described [14]. Membranes were immunoblotted with anti-EMMPRIN/CD-147 HIM6 mAb (BD Biosciences) or anti-uPAR (R&D systems) antibodies. Proteins were visualized with ECL reagent (Pierce) and their relative expression was determined by densitometry using ImageJ software program and normalized relative to β-actin.

### Gelatin and casein-plasminogen zymography

Gelatin or casein-plasminogen zymography was performed as described previously [17]. Serum-free conditioned media were analyzed on 10% SDS-PAGE gels containing either 1 mg/mL gelatin for gelatinase activity or 2 mg/mL casein (Sigma) and 10 μg/mL plasminogen (Calbiochem) for uPA activity.

### Real-time quantitative RT-PCR (qRT-PCR)

Transcript quantification for EMMPRIN, uPA, MMP-2 and MMP-9 was conducted using TaqMan technology and standard curve quantification method. Quantitative PCR were performed using Perfect Master Mix-Probe (AnyGenes, France) on LightCycler system2.0 (Roche, France) according to the manufacturer's techniques. Briefly, cDNAs for EMMPRIN, uPA, MMP-2 and MMP-9 were prepared from total RNA, amplified by RT-PCR, and cloned using TOPO II TA cloning kit (Invitrogen). A standard curve for each transcript was generated using serial dilutions of cloned products ranging from 1 to 10^9 ^molecules/μl. The copy number of unknown samples was calculated by setting their PCR cycle number to the standard curve. Data were normalized to the TBP (TATA-box binding protein) housekeeping gene transcripts. Primers and probes are available under request. All experiments were performed in duplicate.

### Invasion and migration assays

In vitro invasion was assessed using a modified Boyden chamber assay. SCC-9 and DOK cells (2 × 10^4 ^cells/insert for 24-well plate) were plated onto Matrigel-coated cell culture inserts together with either anti-EMMPRIN antibody (20 μg/mL), non-immune IgG, uPA inhibitor amiloride (20 nmol/L; Sigma) or the MMP inhibitor marimastat (10 μmol/L; British Biotechnology, Oxford, UK). Three hours later CHO-Emp mb or CHO mb were added (20 μg/ml). After 48 hours of incubation, cells on the underside of insert filters were fixed, stained with Diff Quik (Dade Behring) and counted under a bright-field microscope. Migration assay was performed as above, omitting matrigel coating.

### Immunofluorescence of cultured cells

DOK and SCC-9 cells were grown in microscope slide chambers to 50-60% confluence. CHO-Emp mb or CHO mb were then added (20 μg/ml) and 24 hours later, cells were washed in PBS and fixed in 4% paraformaldehyde for immunofluorescence detection of EMMPRIN or uPA. Slides were probed with mouse anti-human EMMPRIN antibody (HIM6 BD Pharmingen) or with goat anti human uPA antibody (American diagnostica). After incubation with the corresponding secondary antibody (Alexa 488 and 594) the slides were examined with a laser-scanning confocal microscope (Leica Lasertechnik, Heidelberg). Cells were counterstained with DAPI (Sigma).

### Analysis of human oral squamous carcinoma and dysplastic lesions

Tumor samples were collected from 20 patients with oral squamous carcinoma or dysplastic tissues. **Tissues of normal oral mucosa were collected from healthy donors attending for tooth extraction**. All patients gave their informed consent. Protocols were approved by our institution research ethics committee. Total Formalin-fixed paraffin-embedded (FFPE) tissue sections of **6 normal oral mucosa**, 4 intra epithelial neoplasia, 8 micro-invasive OSCC and 8 invasive OSCC, were analysed for EMMPRIN and uPA expression using qRT-PCR and immunohistochemistry assays. For immunohistochemistry, FFPE sections were stained with a mouse anti-human EMMPRIN antibody (BD-Pharmingen) and a goat anti human uPA antibody (American diagnostica). Staining was visualized using the streptavidin-biotin-HRP detection method, followed by counterstaining with Mayer's hematoxylin. Three-amino-9-ethyl-carbazole (Sigma-Aldrich, France) was used as the chromogen. For transcript quantification levels, RNA was extracted using TRIzol reagent (Invitrogen). First-strand cDNA was synthesized using the High-Capacity cDNA Archive Kit (Applied-Biosystems). Transcript levels were measured in each tissue by qPCR using Perfect Master Mix-Probe (AnyGenes, France) on LightCycler 2.0 System (Roche). The expression levels of interesting transcripts were normalized to the housekeeping TATA-box binding protein (TBP) gene transcripts. All experiments were performed in duplicates and Spearman's rank correlation was used to evaluate the association between mRNA levels in oral dysplastic, micro-invasive and invasive tumor tissues.

### Statistical analysis

Data are expressed as mean F SD. Mann-Whitney and Student's t tests were used to compare differences between groups in various experiments.

## Results

### EMMPRIN and uPA expression is increased with oral tumor progression

**Figure 1:**
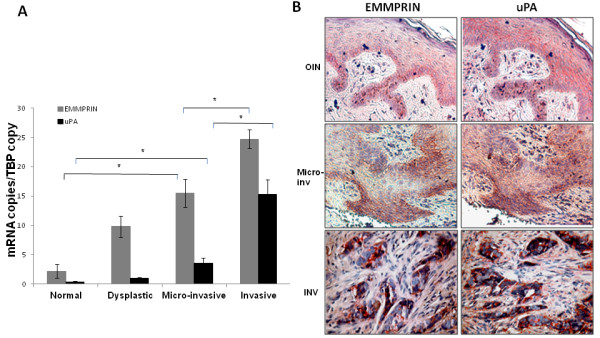
**EMMPRIN and uPA expression in human oral dysplastic and squamous carcinoma lesions**. A- EMMPRIN and uPA mRNA expression in oral dysplastic and tumor tissues. RNA was extracted from formalin-fixed paraffin-embedded **tissue sections of 6 normal oral mucosa (Normal)**, 4 intra epithelial neoplasia (**Dysplastic**), 8 micro-invasive OSCC and 8 invasive OSCC, and were analysed for EMMPRIN and uPA expression using qRT-PCR assays. * denotes significant difference with *p *< 0.05. **B- **Immunohistochemical staining of EMMPRIN and uPA in sections of human oral dysplastic, micro-invasive and invasive squamous carcinoma lesions. Tissue sections from patients with oral dysplastic lesions (OIN), micro-invasive lesions (Micro-inv) and invasive oral squamous carcinoma lesions (INV) lesion sections were subjected to single-labeled immunohistochemistry using mouse anti-human EMMPRIN mAb or goat anti-human uPA Ab and counterstained with HES. EMMPRIN and uPA were colocalized, their staining was heterogeneous with particularly high staining at some regions. Staining was more intense in the tumor sections.

The expression of EMMPRIN and uPA was examined in a tumor series from 20 patients with dysplasctic (4) micro-invasive (8) or invasive lesions (8) **and compared with to their expression in normal oral mucosa (6)**. QRT-PCR analysis has shown that both EMMPRIN and uPA transcripts were proportionally increased with the invasive stage of the tumors (Figure 1A). An average increase of 2.5 fold for EMMPRIN and 11 fold for uPA can be noted between the dysplastic and the invasive stage, **while their levels in normal mucosa were the lowest, displaying 5 fold less EMMPRIN and 3 fold less uPA than in the dysplastic lesions**.

EMMPRIN and uPA expression was also evaluated by immunohistochemical analysis of tumors obtained from OSCC patients. Figure 1B presents a representative staining pattern showing more intense staining of both EMMPRIN and uPA in burgeoning (micro-invasive) and invasive tumor lesions than dysplastic lesions. Moreover, EMMPRIN and uPA staining was localized in the same areas within the tissue.

### EMMPRIN regulates uPA in oral dysplastic DOK and tumoral SCC-9 cell lines

**Figure 2:**
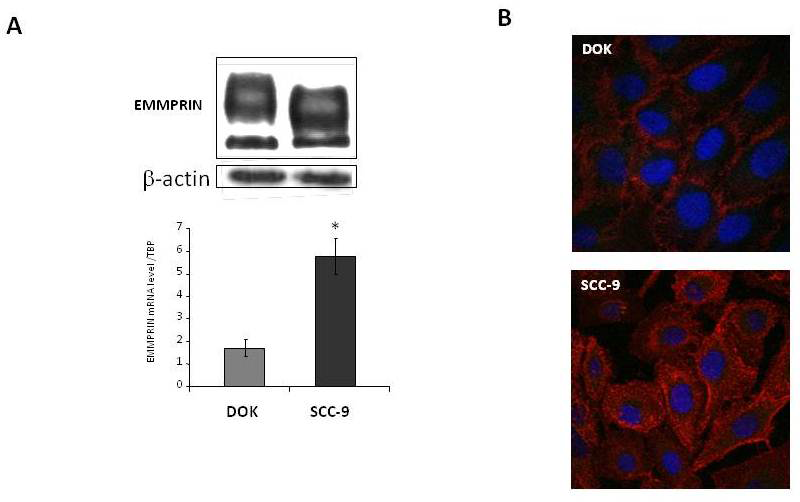
**EMMPRIN expression of dysplastic and tumor cell lines**. **A**, EMMPRIN expression was evaluated by western blotting analysis of 10 μg of DOK and SCC-9 cell lysates (β-actin was used as loading control). Representative blot is shown. EMMPRIN transcripts in DOK and SCC-9 cells were quantified using quantitative RT-PCR. Columns represent average values from at least three independent experiments carried out in triplicate; *bars*, SD. * denotes significant difference with *p *< 0.05. **B**, Immunofluorescence staining for EMMPRIN in DOK and SCC-9 cells. Cells were counterstained with DAPI (×400). EMMPRIN staining was stronger in the SCC-9 tumor cells compared to the Dysplastic DOK cells.

The association of both EMMPRIN and uPA transcript with oral tumor progression and their colocalization within tumor tissues prompted us to look for a regulation of uPA by EMMPRIN. For that, two cell lines, DOK and SCC-9, representing the dysplastic and the invasive stages respectively were studied. These two cell models present a differential expression of EMMPRIN (Figure 2), with SCC-9 showing higher transcript and protein levels of EMMPRIN compared to DOK cells, evaluated by quantitative RT-PCR, western blot and Immunofluorescence analyses.

**Figure 3:**
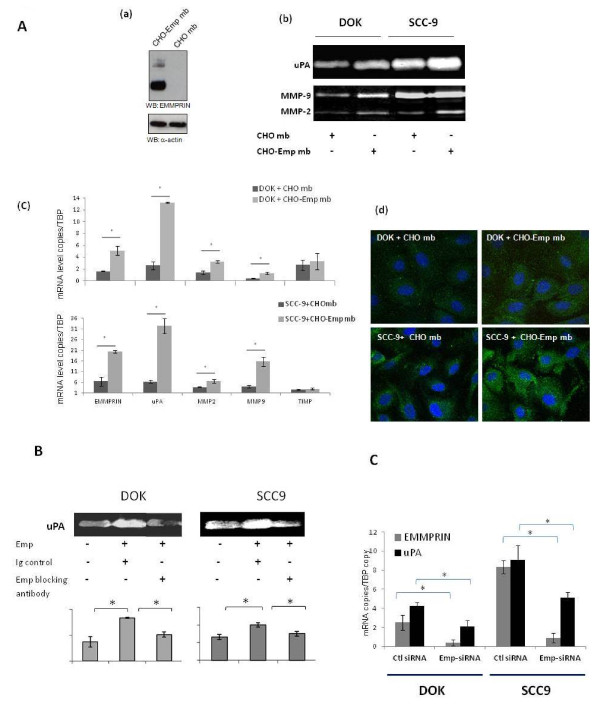
**EMMPRIN regulates uPA in oral dysplastic DOK and tumor SCC-9 cell lines**. A, **(a) **CHO cells were transfected with EMMPRIN cDNA as previously described [18]. Membranes were isolated from CHO-Emp and CHO control cells (CHO-Emp Mb and CHO Mb, respectively) by differential centrifugation and 10 μg of membrane extract were analyzed for EMMPRIN content by immunoblotting. **(b) **DOK and SCC-9 cells (80% confluence) were incubated with 20 μg/mL CHO Mb or CHO-Emp Mb in serum-free medium for 24 h and the conditioned medium was analyzed for uPA activity using casein-plasminogen zymography and for gelatinase activities of MMP-2 and MMP-9 using gelatin zymography (one representative zymography of three independent experiments). **(c) **EMMPRIN, uPA, MMP-2, MMP-9 and TIMP-1 transcripts were quantified using quantitative RT-PCR in DOK and SCC-9 cells incubated for 24 h with CHO Mb or CHO-Emp Mb (20 μg/mL). Columns are means of gene expression relative to TBP housekeeping gene of at least three independent experiments carried out in triplicate; *bars*, SD. * denotes significant difference with *p *< 0.05. (d) SCC-9 and DOK cells were grown in microscope slide chambers to 50-60% confluence and incubated with CHO Mb or CHO-Emp Mb (20 μg/mL). After 24 h, cells were immunostained for uPA using a goat anti-human antibody (green) and counterstained with DAPI (blue). A more intense staining was observed in CHO-Emp mb treated DOK and SCC-9 cells compared to the control CHO mb treated cells. **B**, SCC-9 and DOK cells were incubated with 20 μg/mL of an anti-EMMPRIN blocking antibody (Ancell, Bayport, MN) or with a non-immune IgG antibody in serum-free medium. After 24 h incubation, conditioned medium was harvested for gelatinase and uPA casein zymography. Columns represent average values of the densitometric quantification from at least three independent experiments **carried out in triplicate; *bars*, SD**. * denotes significant difference with *p *< 0.05. **C**, DOK and SCC-9 cells were transfected with EMMPRIN siRNA (Emp-siRNA) or scrambled control siRNA (Ctl siRNA) at 33 nmol/L concentration. EMMPRIN and uPA mRNA expression was evaluated by quantitative RT-PCR analyses. Columns represent mean ± SD of relative expression to TBP housekeeping gene of at least 3 independent experiments carried out in triplicate; *bars*, SD. * denotes significant difference with *p *< 0.05.

The role of EMMPRIN in the regulation of uPA production in these two cell lines was investigated by treating the cells with exogenously added EMMPRIN. EMMPRIN contained within membrane vesicles was obtained from CHO cells previously transfected with EMMPRIN full length cDNA. The incubation of DOK and SCC-9 cells with 20 μg/ml of EMMPRIN-containing membranes (designated CHO-Emp) increased uPA protease activity (by 40% for DOK and 26% for SCC-9) and RNA levels (5 fold for DOK and SCC- 9) when measured by casein-plasminogen zymography and qRT-PCR respectively, whereas those prepared from mock-transfected CHO cells (CHO) had no effect (Figure 3A). This was also accompanied by an increase in MMP-2 and MMP-9 measured by qRT-PCR and gelatine zymography. TIMP-1 which was shown not to be an EMMPRIN target [28] was used here as negative control showing no regulation by EMMPRIN in our cell models (Figure 3Ac). The regulation of uPA by EMMPRIN in both cell lines was confirmed by immunofluorescence analysis (Figure 3Ad).

To evaluate the specificity of EMMPRIN's effects, blocking anti-EMMPRIN antibody was added with the EMMPRIN containing membranes and the effect on uPA production was examined by zymography. As shown in Figure 3B, anti-EMMPRIN antibody (20 μg/mL), but not IgG control, blocked EMMPRIN's effect on uPA in both DOK and SCC-9 cells. A down-regulation of MMP-2 and -9 activities was also observed (data not shown).

The regulation of uPA by EMMPRIN was also investigated by inhibiting endogenous EMMPRIN expression using RNA interference strategy. EMMPRIN siRNA transfection of DOK and SCC-9 cells showed a significant reduction in the transcript level of uPA (Figure 3C).

Hence, EMMPRIN regulated uPA in both the dysplastic and the invasive tumor cell lines, suggesting a regulatory mechanism involved even at the early tumorogenesis stages

### EMMPRIN promotes in vitro cell invasion in oral tumor progression model

**Figure 4:**
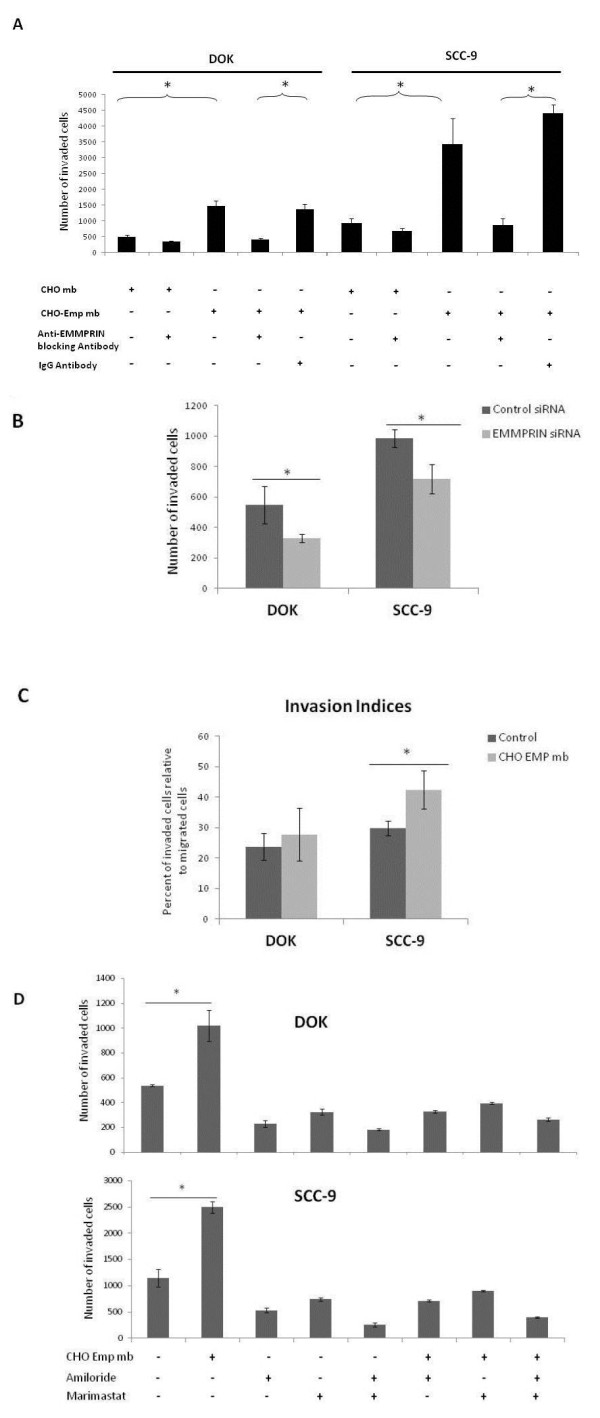
**EMMPRIN regulates invasion in oral dysplastic DOK and tumor SCC-9 cell lines in an MMP and uPA dependent manner. A**, The in vitro invasive property of DOK and SCC-9 cells incubated with CHO-Emp membranes and treated or not with an anti-EMMPRIN blocking antibody (20 μg/mL) were compared using tissue culture Transwell inserts (8-mm pore size; BD Biosciences) placed in a 24-well culture plate. Cells incubated with CHO control or treated with an anti-IgG antibody were used as control. Cells (1 × 10^5^) suspended in serum-free media were seeded into the upper well of each insert onto membranes coated with growth factor-reduced Matrigel (BD Biosciences). After 48 h incubation, cells that remained in the top compartment were removed by cotton swabs, and cells on the underside of insert filters were fixed, stained, and counted under a microscope. The columns shown represent average values from at least three independent experiments carried out in triplicate; *bars*, SD. * denotes significant difference with *p *< 0.05. **B**, DOK and SCC-9 cells were transfected with EMMPRIN siRNA or scrambled siRNA (Ctl siRNA) prior to invasion assays. The columns represent means of three independent experiments carried out in triplicate; *bars*, SD. **C**, Invasive Indices. DOK and SCC-9 cells (1 × 10^5^) seeded into the upper well of tissue culture Transwell inserts coated with growth factor-reduced Matrigel (for invasion assay) or not coated (for migration assay) were incubated with CHO or CHO-Emp membranes (Control and CHO Emp mb respectively). After 48 h of incubation, migrating or invading cells on the underside of insert filters were fixed, stained, and counted under a microscope. Invasion Indices were calculated as percent of the invaded cells relative to the migrated cells. The columns shown represent average values from at least three independent experiments carried out in triplicate; *bars*, SD. * denotes significant difference with *p *< 0.05. **D**, Relative contribution of uPA and MMPs to the in vitro invasive property of DOK and SCC-9 cells. Cells (1 × 10^5^) were seeded into the upper well of matrigel coated inserts and incubated with CHO or CHO-Emp membranes. uPA inhibitor amiloride (20 nmol/L) or MMP inhibitor marimastat (10 μmol/L) were added alone or in combination together with the membranes. After 48 h invading cells were fixed, stained, and counted. Columns represent average values from at least three independent experiments carried out in triplicate; *bars*, SD. * denotes significant difference with *p *< 0.05.

EMMPRIN has already been implicated in tumor cell invasion [10,29]. This was confirmed in our in vitro experiments using the Matrigel invasion assay, showing an increase in the invasive capacity of both cells, DOK and SCC-9, which was abolished by the presence of 20 μg/mL EMMPRIN blocking antibody. The rate of invasion of SCC-9, the more aggressive cell type, was greater than that of DOK. In addition, EMMPRIN increased invasion in both cell types by approximately 3 fold, progressing the invasiveness of the dysplastic cells DOK to that of the invasive SCC-9 cells (Figure 4A). It is interesting that EMMPRIN antibody also inhibited the invasion of control cells (treated with CHO-Mock), suggesting that endogenously produced EMMPRIN also contributes to invasion. Cell invasion was also reduced in both cell lines after inhibition of EMMPRIN by siRNA (Figure 4B).

EMMPRIN also had a more significant effect on the Invasion Indices (calculated as percent of invaded cells relative to migrated cells) of SCC-9 compared to DOK. This emphasizes the protease inducing function of EMMPRIN in the invasion process (Figure 4C).

Since EMMPRIN is mostly known to upregulate MMPs, we investigated the relative contribution of the uPA and MMP systems to the invasion capacity of both cell lines. For that, we used 20 nmol/L amiloride (for uPA inhibition) and 10 μmol/L marimastat (for a global MMP inhibition) which were added alone or in combination. As expected, an inhibition of invasion was observed in both cell lines in the presence of either amiloride or marimastat, the inhibition was greater when the inhibitors were used in combination. However, EMMPRIN treatment did not restore invasion in any of these conditions (Figure 4D). These results suggest that EMMPRIN regulates in vitro oral tumor cell invasion through both uPA and MMP activities.

## Discussion

EMMPRIN is widely distributed in many human and mammalian cells, with notably high expression in some of solids cancers including oral squamous cell carcinoma. It's over-expression is associated with malignancy and ⁄ or poor prognosis in many tumor types, and several studies suggest that EMMPRIN is an important factor which contributes to oral squamous cell carcinoma growth, invasion, and metastasis [30]. EMMPRIN increased expression was observed at a very early stage of oral carcinogenesis. Indeed, it was shown to increase in dysplastic leukoplakias spreading to more superficial layers, and its expression levels correlated significantly with the degree of dysplasia [24].

The functional importance of EMMPRIN during tumor progression has been related mainly to its ability to promote tumor cell invasion by stimulating MMP and VEGF cytokine production expression [31]. We have previously showed that EMMPRIN can also stimulate the serine uPA proteinase system in breast cancer, thus representing an additional degradation pathway enhancing its tumor invasion potential [18]. Our results showing that EMMPRIN also regulated uPA in oral tumors and colocalized with it in the tumoral tissues where both EMMPRIN and uPA were overexpressed suggest that this pathway may play an important role in oral tumor progression. In addition, amiloride, a uPA inhibitor significantly reduced invasion of two oral tumoral cell lines used in this study, DOK and SCC-9, representing the dysplastic and the invasive stages respectively. The fact that combining amiloride with marimastat, an MMPs inhibitor, caused a greater inhibition of EMMPRIN-mediated invasion than either of these inhibitors alone suggests that both pathways function in parallel and underscores EMMPRIN's ability to regulate invasion through both uPA and MMPs. EMMPRIN was shown to be without effect on the MMP inhibitors TIMPs so the induction of MMPs is likely to cause a direct increase in proteolysis [28]. Whether the increase in uPA by EMMPRIN in OSCC is accompanied by a parallel regulation of its inhibitor PAI1 remains to be determined, but the inhibition by amiloride of the increased invasion following EMMPRIN treatment suggests a net increase in the active uPA.

The MAP kinase P38 was implicated in the regulation of uPA, certain MMPs and EMMPRIN itself and may represent a common mechanism for their regulation [7]. As EMMPRIN was suggested to serve as its own receptor in neighbouring cells, it is possible that constitutive EMMPRIN acts as a receptor to the exogenous membrane bound EMMPRIN, initiating signalling pathways that result in P38 activation.

Silencing EMMPRIN in head and neck squamous carcinoma (HNSCC) cells was shown to result in significant suppression of tumor growth [32]. Furthermore, a correlation between the invasive activity and the transcriptional activation of the *uPA *gene has already been reported in oral tumors and cultured OSCC cells and tumors with high levels of both uPA and uPAR were shown to be more invasive [20,33,34]. Correspondingly, our results demonstrate that the more invasive OSCC express more EMMPRIN and more uPA than the dysplastic oral tissues. This was also observed when comparing the corresponding cell lines. This suggests that EMMPRIN levels determine the invasive potential through the regulation of the two proteolytic systems, MMPs and uPA; the increase in EMMPRIN expression during progression upregulates protease production and promotes greater invasion. Indeed, when the less invasive DOK cells were treated with exogenous EMMPRIN, their invasiveness approached that of the SCC9 invasive cells.

## Conclusions

Although the role of EMMPRIN in tumor growth and metastasis has been studied in detail, the mechanisms by which it exerts its effects in OSCC tumorogenesis are not completely understood. Our results showing the induction of the uPA system provide important insights into the mechanism by which EMMPRIN contributes to oral tumor cell invasion and progression.

## Conflict of interests

The authors declare that they have no competing interests.

## Authors' contributions

**GL**: carried out in vitro experiments; **SM (S Menashi)**: participated in the design of the study, data analyses and writing of the manuscript; **BCB**: coordinated tissue specimens study and analyzed Immunohistochemistry data; **FK**: carried out experiments; **CQ**: carried out experiments; **MPP**: carried out qPCR analyses; **BN**: developed analytical tools; **FC**: participated in the design of the study and research discussions; **CL**: participated in the design of the study and data analyses; **SM (S Mourah)**: participated in the design of the study, statistical and data analysis and writing of the manuscript. All authors read and approved the finalmanuscript.

## Pre-publication history

The pre-publication history for this paper can be accessed here:

http://www.biomedcentral.com/1471-2407/12/115/prepub
